# Evidence-based psychosocial interventions in schizophrenia: a critical review

**DOI:** 10.1097/YCO.0000000000000925

**Published:** 2024-02-15

**Authors:** Stefano Barlati, Gabriele Nibbio, Antonio Vita

**Affiliations:** a Department of Clinical and Experimental Sciences, University of Brescia; b Department of Mental Health and Addiction Services, ASST Spedali Civili of Brescia, Brescia, Italy

**Keywords:** evidence-based, functioning, psychosocial interventions, rehabilitation, schizophrenia

## Abstract

**Purpose of review:**

Schizophrenia Spectrum Disorders (SSD) are severe conditions that frequently produce significant impairment in cognitive performance, social skills and psychosocial functioning. As pharmacological treatment alone often provides only limited improvements on these outcomes, several psychosocial interventions are employed in psychiatric rehabilitation practice to improve of real-world outcomes of people living with SSD: the present review aims to provide a critical overview of these treatments, focusing on those that show consistent evidence of effectiveness.

**Recent findings:**

Several recent systematic reviews and meta-analyses have investigated in detail the acceptability, the effectiveness on several specific outcomes and moderators of response of different psychosocial interventions, and several individual studies have provided novel insight on their implementation and combination in rehabilitation practice.

**Summary:**

Cognitive remediation, metacognitive training, social skills training, psychoeducation, family interventions, cognitive behavioral therapy, physical exercise and lifestyle interventions, supported employment and some other interventions can be fully considered as evidence-based treatments in SSD. Psychosocial interventions could be of particular usefulness in the context of early intervention services. Future research should focus on developing newer interventions, on better understanding the barriers and the facilitators of their implementation in clinical practice, and exploring the opportunities provided by novel technologies.

## INTRODUCTION

### Background

Schizophrenia Spectrum Disorders (SSD) represent severe and debilitating mental conditions, frequently characterized by impaired cognitive performance [1,2], poor real-world functional outcomes [3,4], reduced quality of life [5,6], high levels of internalized stigma [7–9] and low levels of life engagement [10,11]. In people living with SSD, a combination of reduced access to medical care, unhealthy lifestyles and biological factors lead to an average reduction of life expectancy of 14.5 years, mainly due to cardiovascular disease and cancer [12^▪▪^,^13^].

Pharmacological treatment represents the cornerstone of SSD treatment, and indeed a massive body of evidence reports that antipsychotic medications are consistently effective in improving psychotic symptoms, preventing relapses and even extending life expectancy in people living with SSD [14–16]. However, pharmacological treatment alone is not currently effective in improving several clinical and functional outcomes, such as cognitive performance, social skills and quality of life, and in improving real-world outcomes, such as finding and maintaining a job or having meaningful personal relationships; in fact, most people living with SSD currently experience only small improvements in outcomes that are important for them in their personal perspective and do not achieve full functional and personal recovery [17–19].

This is where psychosocial interventions come into play. Complementing and enhancing the effects of pharmacological treatments, and targeting domains and features that are not currently improved by antipsychotic treatment, various psychosocial interventions have shown consistent effectiveness on several different outcomes [20^▪^,^21^^▪▪^], and are now recommended as evidence-based treatments for SSD in many national and international guidelines [14,22–24,25^▪^].

Considering that SSD represent a clinically heterogeneous spectrum and no valid one-size-fits-all treatment protocol exists, having a good understanding of the different available evidence-based psychosocial interventions is essential to devise and implement personalized treatment programs, with specific interventions for the specific needs of specific patients [18]: this currently represents a fundamental step to provide the most effective treatment for people living with SSD. 

**Box 1:**
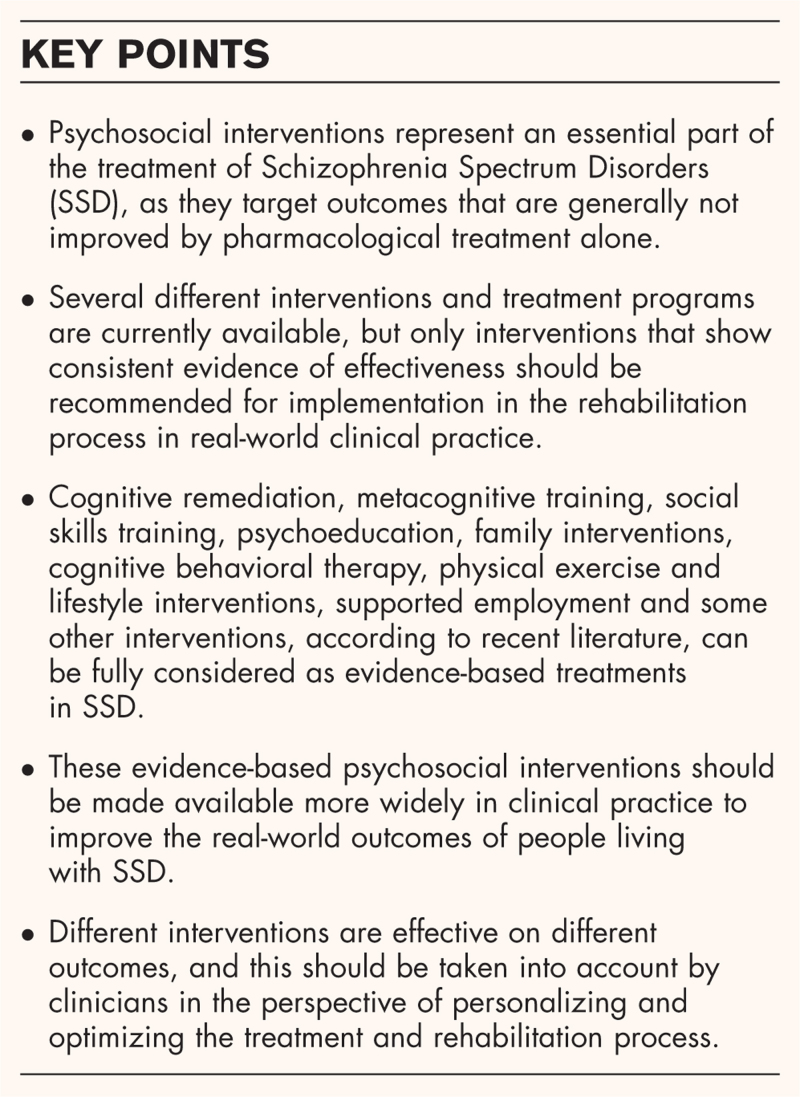
no caption available

### Aims

Rather than providing an assessment of the overall effectiveness of psychosocial interventions in SSD, the present work will focus on each specific evidence-based psychosocial intervention, reporting and commenting the available and recent evidence regarding its effectiveness on global as well as on specific outcomes. A summary is reported in Table 1. Discussion regarding the gaps in current scientific literature and the intrinsic limitations of specific psychosocial interventions, as well as considerations on the current state of the art and on the implementation of these interventions in clinical practice will also be provided.

**Table 1:** Evidence-based psychosocial interventions in Schizophrenia Spectrum Disorders

Intervention	Definition	Main outcomes	Systematic evidence of effectiveness
Cognitive Remediation	Behavioral training-based intervention targeting cognitive performance.	Cognitive performance, with the aim of providing a durable improvement to psychosocial functioning.	Cognitive performance and psychosocial functioning [28–30], acceptability [31^▪^].
Metacognitive Training	Intervention combining elements of psychoeducation, cognitive bias modification and strategy teaching targeting metacognition.	Metacognition, with the aim of improving, positive symptoms, persistent symptoms, insight and psychosocial functioning.	Positive symptoms and psychosocial functioning [34], insight [35^▪^].
Social Skills Training	Training intervention that targets interpersonal and social skills.	Social skills and social functioning, with the aim of improving real-world outcomes such as social performance and social interactions	Social performance outcomes, clinical symptoms [37,38].
Psychoeducation	Interventions focused on the education of an individual living with a psychiatric disorder on the topics concerning the disorder itself.	Relapse prevention and treatment adherence, aiming at the improvement of psychosocial functioning.	Relapse prevention [47], psychosocial functioning [46], also in clinical high risk individuals [48^▪^].
Family Interventions	Interventions including family members of individuals living with mental disorders, conducted with or without the patient, often including elements of psychoeducation.	Family education and management of the disorder, aiming to improve relapse prevention treatment adherence, psychosocial functioning.	Relapse prevention [52^▪^], family level and patient-level psychological well being outcomes [53].
Cognitive Behavioral Therapy for Psychoses	Structured psychotherapy intervention focusing on the connections between thoughts, behaviors, and emotions, targeted and adapted for the treatment of psychotic conditions	Positive and negative symptoms, and persistent symptoms more in general, aiming at the improvement of several real-world outcomes.	Positive symptoms [56^▪▪^], clinical symptoms and psychosocial functioning [57^▪^], transition to psychosis in at-risk subjects [58^▪^].
Physical Exercise and Lifestyle Interventions	Interventions including elements of physical training, often aerobic exercise, and interventions modifying unhealthy lifestyle habits.	Physical fitness, metabolic and health-related outcomes, but in people living with mental disorders also cognitive performance, symptoms severity and psychosocial functioning.	Metabolic and health related outcomes [61], cognitive performance [63^▪^], clinical symptoms and psychosocial functioning [66^▪▪^].
Supported Employment	Interventions combining different professional figures in order to assist participants with obtaining and maintaining employment.	Real-world work-related outcomes such as obtaining and maintaining in a stable manner an employment and acquiring and improving professional skills.	Employment related outcomes such as employment rate, job duration and wages [68^▪^].

## COGNITIVE REMEDIATION

Cognitive Remediation (CR) is a behavioral training-based intervention targeting cognitive performance with the aim of providing a durable improvement to psychosocial functioning [26,27]. It currently represents the psychosocial intervention with the highest degree of recommendation in the European Psychiatric Association guidelines for the treatment of cognitive impairment in schizophrenia [25^▪^].

Two high-quality meta-analyses have recently explored the effectiveness of CR, one including both interventions targeting neurocognitive performance and interventions targeting social cognition [28], and one considering only neurocognition-targeting programs [29]. Both meta-analyses yielded very similar results, showing that CR provided significant benefits in global cognitive performance as well as in specific cognitive domains that were translated into significant improvement in psychosocial functioning. The effectiveness of social cognition training was also explored in a dedicated meta-analysis, reporting significant improvements in social cognition domains and generalization to the executive functions neurocognitive domain [30].

Considering treatment-related moderators of effect, the presence of an active and trained therapist delivering the intervention, the structured development of novel cognitive strategies, the implementation of techniques to transfer cognitive gains into the real world and the integration with structured psychiatric rehabilitation programs or other evidence-based psychosocial interventions significantly improved outcomes: these factors emerged as core treatment ingredients, and programs including all these elements provided moderate-sized effects on both global cognition and psychosocial functioning. As regards participant-related predictors of response, no specific characteristics represented a barrier to effectiveness, but more clinically compromised participants reported greater improvements [28].

The acceptability of CR interventions was also systematically assessed: a recent meta-analysis investigated CR trials drop-outs, and found that CR overall has a good acceptability profile, in line with that of other psychosocial interventions [31^▪^]. Evidence from low-income settings also suggest that CR can be feasible and implemented in clinical practice also with very limited available resources [32].

The main limitation of CR interventions is that, on themselves, they provide no substantial benefits as regards psychotic symptoms. The results of an earlier meta-analysis suggested that CR can provide improvements in negative symptoms [33], but more recent meta-analyses including more high quality studies reported that these gains, if statistically significant, are too small sized to be of clinical relevance [28,29].

## METACOGNITIVE TRAINING

Metacognitive training for psychosis (MCT) is a psychosocial intervention that combines elements of psychoeducation, cognitive bias modification and strategy teaching, aiming at improving positive symptoms, and persistent symptoms more in general, by improving metacognitive function; it represent the most employed and most investigated metacognitive intervention, a group of treatments that also includes metacognitive therapy and metacognitive insight and reflection therapy [34].

A recent and high-quality meta-analysis explored the effectiveness of MCT on several different outcomes: MTC provided significant long-term improvement in positive symptoms, particularly delusions, and psychosocial functioning; significant, albeit smaller effects were also observed in negative symptoms, cognitive biases and self-esteem [35^▪^].

Another meta-analysis investigated the effectiveness of metacognitive interventions on insight: MCT improved self-reflectiveness and overall cognitive insight both after treatment and at follow-up observations, and self-certainty after treatment only. Findings on clinical insight could not be quantitatively synthesized, but trials results suggest that MCT can be effective also in this aspect [36].

## SOCIAL SKILLS TRANING

Social skills training (SST) is a psychosocial intervention that targets interpersonal and social skills with the aim of improving real-world outcomes such as social performance and social interactions. Meta-analytic evidence shows that SST provides improvements in social outcomes as well as significant albeit small improvements in negative and general psychopathology symptoms [37,38].

As the overall effectiveness of SST in SSD has already been well documented and established for several years [39], recent studies have focused in on combining SST with other psychosocial interventions, in particular components of cognitive behavioral psychological interventions, CR and MCT, showing positive synergies on different outcomes with these combined treatments [40–44].

## PSYCHOEDUCATION

Psychoeducation encompasses all the interventions focused on the education of an individual living with a psychiatric disorder regarding topics that may improve the outcomes of treatment and rehabilitation, enabling a behavioral change in the participant; in the treatment of SSD, psychoeducation has been recognized since several years as an intervention that can consistently improve relapse prevention and treatment adherence [45], and some evidence also suggests that it can improve psychosocial functioning and some psychopathological domains, albeit not core SSD symptoms [46]. A recent and high-quality network meta-analysis exploring the effectiveness of different psychosocial interventions on relapse prevention confirmed that psychoeducation has a good effectiveness on this specific outcome; this positive effect however was not observed at follow-up observations longer than 12 months [47].

A recent systematic review explored the effects of psychoeducation on individuals at clinical high risk for psychosis: the results highlighted a good feasibility and acceptability profile of the interventions in this population, and some studies also reported positive effects on psychosocial functioning and psychopathological outcomes, but more high-quality research is currently needed to evaluate the effectiveness of psychoeducation in this population, particularly on high-relevance outcomes such as transition to psychosis [48^▪^].

## FAMILY INTERVENTIONS

It has been widely demonstrated that family environment plays a pivotal role in the long-term course of SSD, as well as in the recovery process [49]. In this context, several different family interventions models have been developed [50,51].

A recent high-quality network meta-analysis explored the effectiveness of different family interventions in relapse prevention: the vast majority of interventions included some element of family psychoeducation, and almost all interventions were effective in preventing relapse even at follow-up observations longer than 12 months; family psychoeducation alone emerged as the most effective intervention, superior to more complex models that include other treatment elements and showing a moderate-to-large effect size, while the less effective approach were community-based interventions involving family members [52^▪^].

Another recent meta-analysis explored and attested the effectiveness of family interventions on several different family-level (family's mental health, attitude towards the disorder, family burden, family coping, family health and well being, family functioning) and patient-level (treatment satisfaction and adherence, quality of life, psychiatric symptoms, illness insight, psychosocial functioning, rehospitalization) outcomes: moderate-to-large effect sizes were observed in both categories, with superior effects in family outcomes. Interventions targeting individual family units and delivered only to the family caregivers emerged as superior. The results of this meta-analysis, however, have to be considered with caution as significant publication bias was reported [53].

Overall, family interventions appear to represent one of the most clinically meaningful categories of psychosocial interventions, but to date the number of studies exploring their effectiveness is still somehow limited, compared to that available for other psychosocial interventions: in this regard, more research on this field is warranted.

## COGNITIVE BEHAVIORAL THERAPY

Cognitive Behavioral Therapy for psychosis (CBTp) is a structured psychotherapy intervention that focuses on the connections between thoughts, behaviors, and emotions targeted and adapted for the treatment of SSD. It represents an evidence-based psychotherapy intervention that has been shown to be effective in improving several outcomes, and in particular in reducing the severity of positive symptoms [54,55].

A recent umbrella review of meta-analyses and randomized controlled trials showed a consistent effectiveness of CBT positive symptoms, which represents one of its primary outcomes, while small and nonconsistent effects were observed for negative symptoms [56^▪▪^].

A recent meta-analysis investigated the effectiveness of CBTp delivered in a group setting: the results of this work partially contested those of previous meta-analyses, showing no significant benefit as regards the severity of positive and negative symptoms, but reported positive effects on other important outcomes such as psychosocial functioning and global psychopathological severity [57^▪^].

Another recent meta-analysis investigated the use of CBTp in the prodromal phases of psychosis: the results showed that this intervention is indeed effective in reducing the transition to full psychosis at all considered time-points and also in reducing attenuated psychotic symptoms [58^▪^]. These results are very interesting in a clinical perspective, as this population may represent a target that benefits in particular manner for CBTp, with significant and important long-term consequences.

## PHYSICAL EXERCISE AND LIFESTYLE INTERVENTIONS

Physical exercise can be considered to all intents and purposes as a fully evidence-based psychosocial intervention for people living with SSD, capable of improving not only physical fitness, but also psychopathological outcomes [59] and cognitive performance [60–62].

A recent and large meta-analysis focused on moderators of effects of cognitive improvement, and confirmed that the most effective form of physical exercise for this outcome is aerobic exercise; it also reported a superior effect of group exercise, that supervision of trained exercise professionals substantially enhanced effectiveness and that positive results could be observed with a dose-dependent effect starting from a duration of ≥90 min per week for ≥12 weeks [63^▪^]. Recent evidence also suggest that combining physical exercise with CR produces a synergic effect, providing faster gains in cognitive performance [64,65].

Another recent meta-analysis explored the effectiveness of physical exercise in people living with SSD on psychosocial functioning: positive and moderate-sized effects were observed for global functioning, for social functioning and for daily life functioning [66^▪▪^].

Finally, physical exercise, as well as diet and lifestyle interventions were investigated regarding their effectiveness on several different outcomes: anthropometric measures such as BMI weight and waist circumference showed significant lasting benefits, alongside psychopathological, cognitive and functional measure, including quality of life [61]. In this regard, physical exercise and lifestyle interventions represent an intervention that might be suitable for the vast majority of people living with SSD and be particularly useful in cases where targeting cognitive performance represents a priority.

## SUPPORTED EMPLOYMENT

Supported employment and, overall, interventions specifically targeting employment represent a very particular category of psychosocial interventions that, when delivered to people living with SSD, have been show to improve the likelihood of obtaining a competitive job and to improve the number of hours worked in any job [67].

A recent meta-analysis explored the effectiveness of individual placement and support, a rehabilitation program focused on employment outcomes, across all different psychiatric diagnoses: the results showed that the intervention was effective in all the included populations, but it was more effective in people with severe mental illness and with SSD in particular. The effectiveness of the intervention, however, emerged as limited by symptoms severity [68^▪^].

Despite this limitation, the evidence supporting the usefulness of this approach is consistent, and is recently leading to the development of novel intervention programs and protocols [69].

In clinical practice, interventions targeting employment may represent a valuable asset to progress in the recovery process of subjects with a stable clinical condition and good cognitive performance, or where clinical recovery and cognitive performance improvement were already obtained.

## OTHER INTERVENTIONS

Several other interventions have been explored in the treatment of different aspects of SSD.

Assertive Community Treatment (ACT) represents an intensive mental health program model including multidisciplinary approaches that can improve clinical and functional outcomes [70]. A recent study has investigate whether a flexible and less resource-demanding format of ACT can be equally effective, but reported negative findings, with the full ACT group emerging as superior on personal and social functioning outcomes [71].

Compensatory interventions for cognitive impairment do not directly target cognitive performance, but rather provide targeted aids and strategies to improve functioning despite cognitive deficits: a meta-analysis exploring the effectiveness of this approach has indeed observed functional improvements that were maintained at follow-up observations [72]. Elements of these interventions could be combined with CR interventions to further increase functioning gains, and they appear to be ideal in participants that do not respond to CR.

Illness self-management interventions, focusing on teaching and training skills to autonomously manage the physical, social and emotional impact of a disorder, provided small but significant improvements in different outcomes in two meta-analyses [73,74].

Motivational interviewing has recently been explored in a meta-analysis in people with SSD and comorbid substance use disorders, reporting mostly negative results [75]. A systematic review investigating the effectiveness on medication adherence was also conducted, again reporting mostly negative findings [76].

Mindfulness-based interventions [77] have also been investigated in people living with SSD, and the results of some studies suggests that they might be effective in improving clinical and functional outcomes [78]; however, the quantity and the quality of the studies investigating this intervention is not currently sufficient to consider it as fully evidence-based.

## EARLY INTERVENTION SERVICES

Early intervention services are designed specifically to provide treatment in first episode or early phase of psychosis subjects, and indeed a wealth of recent literature shows that multidisciplinary teams of mental health professionals providing multimodal treatment in this population produces considerable long-term benefits [79]. In fact, recent high-quality evidence shows that providing evidence-based psychosocial interventions in early phase subjects clearly represents the most cost-effective course of action, and possibly the overall most effective approach [21^▪▪^].

However, implementing early intervention services in routine clinical practice is often accompanied by many challenges, mostly linked to the difficulty of accurately identifying and intercepting early-phase subjects and of building an effective therapeutic alliance with subjects and their families. Organization and resource availably issues might also occur, as maintaining an effective multidisciplinary intervention service might represent a complex endeavor in and of itself [80].

## CONLCUSIONS AND FUTURE DIRECTIONS

Several different psychosocial interventions for people living with SSD have shown consistent evidence of effectiveness in different clinically and personally relevant outcomes.

Most interventions have shown a measure of effectiveness on psychosocial functioning outcomes, and most people living with SSD, despite the recommendations provided in national and international guidelines, at the present time receive only pharmacological treatment [81]. In this perspective, most people living with SSD would currently benefit in a considerable manner from receiving any kind of evidence-based psychosocial intervention.

However, in the perspective of personalizing and optimizing the treatment options, improving the chances of recovery and accelerating the recovery process [18], identifying the most appropriate intervention for each individual, and even the most appropriate intervention for the specific phase of the illness and of the recovery journey, actually represents the optimal approach.

CRT and physical exercise are particularly effective in improving cognitive performance: they could be useful in the vast majority of patients, and particularly in those that show cognitive impairment.

Physical exercise may also be particularly useful in subjects showing metabolic issues and medication -related metabolic adverse effects [82,83]. CBTp may also be useful in most patients, and, as MCT, may help in improving positive symptoms that persist with pharmacological treatment. Family interventions and individual psychoeducation could also be of use in the vast majority of patients but may provide the most important results in people with multiple or frequent relapses. SST may be combined with most other interventions to further improve functioning and be suited to individuals with social skills deficits. Finally, supported employment could be of use in individuals with less severe symptoms and smaller clinical impairment, or individuals that have already regained more basic skills and abilities.

It is also important to note that combining different interventions often produces synergic effects, so integrating interventions often represents an effective strategy if the available resources allow this approach [28,43,64].

Future research should focus on developing newer, more effective and more optimized interventions and treatment programs, but also on better understanding the barriers and the facilitators of the implementation in real-world everyday clinical practice of evidence-based interventions, aiming to further reduce and resolve the bench-to-bedside gap [84,85].

Finally, research on the usefulness of new digital technologies, including telemedicine and immersive virtual reality approaches, to deliver evidence-based interventions [86–89] could open new avenues and perspective to improve the recovery process of people living with SSD.

## Acknowledgements

*None.*

### Financial support and sponsorship

*None.*

### Conflicts of interest

*There are no conflicts of interest.*

## REFERENCES AND RECOMMENDED READING

Papers of particular interest, published within the annual period of review, have been highlighted as:

▪ of special interest
▪▪ of outstanding interest
